# Supplementary material for: Pyruvate Dehydrogenase Kinase 1 inhibition mediated oxidative phosphorylation enhancement in cartilage promotes osteoarthritis progression
Source: BMC Musculoskelet Disord. 2023 Jul 20;24:597. doi: 10.1186/s12891-023-06585-6 (PMC10357736; doi:10.1186/s12891-023-06585-6)
Supplement: Supplementary file 1 — Additional file 1. [file 12891_2023_6585_MOESM1_ESM.zip › MF.Gsea.1653627316242/GOMF_LOW_DENSITY_LIPOPROTEIN_PARTICLE_RECEPTOR_BINDING.html]

Details for gene set GOMF\_LOW\_DENSITY\_LIPOPROTEIN\_PARTICLE\_RECEPTOR\_BINDING[GSEA]

|  || Dataset | 55457\_collapsed\_to\_symbols.cls.cls #OA\_versus\_normal.cls.cls #OA\_versus\_normal\_repos |
| Phenotype | cls.cls#OA\_versus\_normal\_repos |
| Upregulated in class | OA |
| GeneSet | GOMF\_LOW\_DENSITY\_LIPOPROTEIN\_PARTICLE\_RECEPTOR\_BINDING |
| Enrichment Score (ES) | 0.35159263 |
| Normalized Enrichment Score (NES) | 1.1311737 |
| Nominal p-value | 0.2904762 |
| FDR q-value | 0.6414284 |
| FWER p-Value | 1.0 |
Table: GSEA Results Summary

  

Fig 1: Enrichment plot: GOMF\_LOW\_DENSITY\_LIPOPROTEIN\_PARTICLE\_RECEPTOR\_BINDING      
 Profile of the Running ES Score & Positions of GeneSet Members on the Rank Ordered List

  

| SYMBOL | TITLE | RANK IN GENE LIST | RANK METRIC SCORE | RUNNING ES | CORE ENRICHMENT || 1 | SNX17 | sorting nexin 17 [Source:HGNC Symbol;Acc:HGNC:14979] | 60 | 0.971 | 0.1207 | Yes |
| 2 | LRPAP1 | LDL receptor related protein associated protein 1 [Source:HGNC Symbol;Acc:HGNC:6701] | 375 | 0.716 | 0.1888 | Yes |
| 3 | LDLRAP1 | low density lipoprotein receptor adaptor protein 1 [Source:HGNC Symbol;Acc:HGNC:18640] | 445 | 0.684 | 0.2717 | Yes |
| 4 | AP2M1 | adaptor related protein complex 2 subunit mu 1 [Source:HGNC Symbol;Acc:HGNC:564] | 1419 | 0.441 | 0.2531 | Yes |
| 5 | LANCL1 | LanC like 1 [Source:HGNC Symbol;Acc:HGNC:6508] | 1507 | 0.428 | 0.3015 | Yes |
| 6 | DAB2 | DAB adaptor protein 2 [Source:HGNC Symbol;Acc:HGNC:2662] | 1821 | 0.381 | 0.3265 | Yes |
| 7 | CLU | clusterin [Source:HGNC Symbol;Acc:HGNC:2095] | 2083 | 0.352 | 0.3516 | Yes |
| 8 | APOE | apolipoprotein E [Source:HGNC Symbol;Acc:HGNC:613] | 2776 | 0.272 | 0.3330 | No |
| 9 | SYT1 | synaptotagmin 1 [Source:HGNC Symbol;Acc:HGNC:11509] | 3829 | 0.173 | 0.2738 | No |
| 10 | CLTC | clathrin heavy chain [Source:HGNC Symbol;Acc:HGNC:2092] | 4330 | 0.126 | 0.2512 | No |
| 11 | CRP | C-reactive protein [Source:HGNC Symbol;Acc:HGNC:2367] | 5154 | 0.059 | 0.1949 | No |
| 12 | ANKRA2 | ankyrin repeat family A member 2 [Source:HGNC Symbol;Acc:HGNC:13208] | 5840 | 0.006 | 0.1425 | No |
| 13 | DKK1 | dickkopf WNT signaling pathway inhibitor 1 [Source:HGNC Symbol;Acc:HGNC:2891] | 6392 | -0.034 | 0.1041 | No |
| 14 | PICALM | phosphatidylinositol binding clathrin assembly protein [Source:HGNC Symbol;Acc:HGNC:15514] | 8905 | -0.225 | -0.0618 | No |
| 15 | HSP90B1 | heat shock protein 90 beta family member 1 [Source:HGNC Symbol;Acc:HGNC:12028] | 9134 | -0.242 | -0.0483 | No |
| 16 | HSPG2 | heparan sulfate proteoglycan 2 [Source:HGNC Symbol;Acc:HGNC:5273] | 9449 | -0.269 | -0.0379 | No |
| 17 | DNAJA1 | DnaJ heat shock protein family (Hsp40) member A1 [Source:HGNC Symbol;Acc:HGNC:5229] | 10041 | -0.325 | -0.0418 | No |
| 18 | APBB3 | amyloid beta precursor protein binding family B member 3 [Source:HGNC Symbol;Acc:HGNC:20708] | 11509 | -0.502 | -0.0909 | No |
| 19 | APOB | apolipoprotein B [Source:HGNC Symbol;Acc:HGNC:603] | 11794 | -0.548 | -0.0422 | No |
| 20 | APBB1 | amyloid beta precursor protein binding family B member 1 [Source:HGNC Symbol;Acc:HGNC:581] | 12811 | -0.992 | 0.0069 | No |
Table: GSEA details [plain text format]

  

Fig 2: GOMF\_LOW\_DENSITY\_LIPOPROTEIN\_PARTICLE\_RECEPTOR\_BINDING      
 Blue-Pink O' Gram in the Space of the Analyzed GeneSet

  

Fig 3: GOMF\_LOW\_DENSITY\_LIPOPROTEIN\_PARTICLE\_RECEPTOR\_BINDING: Random ES distribution      
 Gene set null distribution of ES for **GOMF\_LOW\_DENSITY\_LIPOPROTEIN\_PARTICLE\_RECEPTOR\_BINDING**
